# Low-Cost Hyperspectral Imaging System: Design and Testing for Laboratory-Based Environmental Applications

**DOI:** 10.3390/s20113293

**Published:** 2020-06-09

**Authors:** Mary B. Stuart, Leigh R. Stanger, Matthew J. Hobbs, Tom D. Pering, Daniel Thio, Andrew J.S. McGonigle, Jon R. Willmott

**Affiliations:** 1 Department of Electronic and Electrical Engineering, University of Sheffield, Sheffield S1 4DE, UK; mbstuart1@sheffield.ac.uk (M.B.S.); lrstanger1@sheffield.ac.uk (L.R.S.); m.hobbs@sheffield.ac.uk (M.J.H.); 2 Department of Geography, University of Sheffield, Sheffield S10 2TN, UK; t.pering@sheffield.ac.uk (T.D.P.); a.mcgonigle@sheffield.ac.uk (A.J.S.M.); 3 Nunnery Lane Dental Practice, York YO23 1AH, UK; nunnerylanedental@gmail.com; 4 School of Geosciences, University of Sydney, Sydney, NSW 2006, Australia; 5 Faculty of Health, Engineering and Sciences, University of Southern Queensland, Toowoomba, QLD 4350, Australia

**Keywords:** low-cost, miniature sensor, hyperspectral, laboratory-based, environmental monitoring

## Abstract

The recent surge in the development of low-cost, miniaturised technologies provides a significant opportunity to develop miniaturised hyperspectral imagers at a fraction of the cost of currently available commercial set-ups. This article introduces a low-cost laboratory-based hyperspectral imager developed using commercially available components. The imager is capable of quantitative and qualitative hyperspectral measurements, and it was tested in a variety of laboratory-based environmental applications where it demonstrated its ability to collect data that correlates well with existing datasets. In its current format, the imager is an accurate laboratory measurement tool, with significant potential for ongoing future developments. It represents an initial development in accessible hyperspectral technologies, providing a robust basis for future improvements.

## 1. Introduction

The continued development of hyperspectral imaging technologies represents a significant area of study with the potential to revolutionise data acquisition methods across a vast range of research domains. Whilst, traditionally, hyperspectral imaging sensors were based within spaceborne and aircraft set-ups [1,2], the recent surge in miniaturised, low-cost imagers as provides an opportunity to expand the scope of these technologies to a variety of field-based and portable applications [3,4,5,6,7]. These developments not only began to improve on the often substantial costs associated with traditional hyperspectral data collection methods, but they also significantly improved the accessibility of hyperspectral sensing technologies [3,4,8]. These new imagers provide accurate, high-spatial-resolution datasets that are not constrained by factors, such as variable meteorological conditions, limited temporal resolution, and expensive set-up costs, which readily affect traditional monitoring methods [6,9,10]. Furthermore, as these technologies continue to improve and develop, we can expect to see a substantial increase in the availability of hyperspectral datasets across a wide variety of environments.

Hyperspectral imaging was also proven to be a powerful tool for laboratory-based applications, yet the majority of imagers currently used in these settings remain bulky and expensive, subsequently limiting their user base and accessibility [8]. However, as the recent development of low-cost components and electronics enhanced field-based applications, it can also inspire advancement within a laboratory setting. In this article, we introduce a laboratory-based hyperspectral imager that was developed using low-cost, commercially available components, representing a significant reduction in hyperspectral system development costs. Existing hyperspectral cameras on sale today can cost upward of £30,000 (e.g., the Specim FX17e), with some commercial systems costing as much as £150,000 (e.g., the Specim FX50). In comparison, our low-cost hyperspectral imager costs less than £6000 to develop with the main costs associated with the development systems. A critical component used within our imager is the miniature spectrometer. These compact components drive the development of low-cost spectral sensing instrumentation with wavelengths ranging from ultra-violet through to infrared. At present, our imager is capable of detecting wavelengths within the visible portion of the electromagnetic spectrum. However, it could be converted to cover different ranges, such as the infrared, by incorporating a different wavelength miniature spectrometer. This article discusses the initial development and performance testing of this imager within a laboratory setting, providing a metrology-based calibration for the instrument before applying it to a variety of environmental applications to demonstrate its potential as a valuable laboratory measurement tool with the potential to provide significant improvements to the accessibility of robust hyperspectral imaging. The hyperspectral set-up, including component configuration, image capture and correction, and calibration methods, is discussed in Section 2. Section 3 goes on to demonstrate the imager’s abilities in three separate environmental applications. These findings are discussed in the context of the individual applications within Section 3, with a wider summary of the imager’s abilities provided in Section 4.

## 2. Materials and Methods

**Figure 1:**
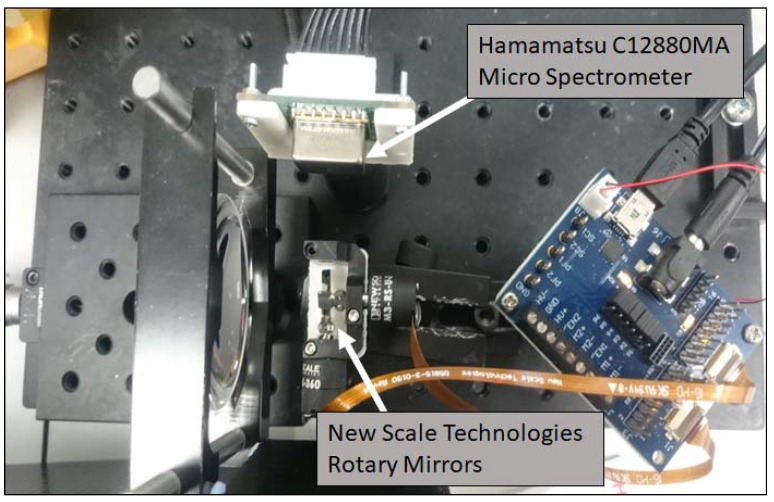
Components of the hyperspectral set-up.

**Figure 2:**
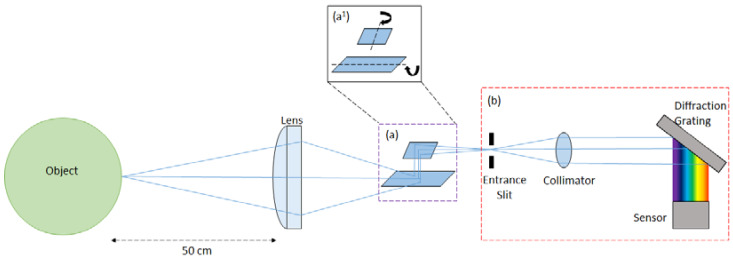
Schematic diagram of the low-cost hyperspectral imager: (**a**) and (**b**) comprise the rotary mirror system and the miniature spectrometer respectively, illustrating the main components of each device. Inset (**a^1^**) displays the rotational axis for each mirror. Note, beam steering of the field of view (FOV) is provided by the mirrors. The image distance is ca. 66.6 mm. This is distributed as follows: ca. 30 mm lens to mirrors, ca. 4.6 mm between mirrors, and ca. 32 mm mirrors to spectrometer. Not to scale.

The developed imager (Figure 1) comprises a basic Thorlabs Plano-convex lens (LA1401-A, Ely, UK), a rotary mirror system, and a miniature spectrometer. Spectral capture is completed using a C12880MA Hamamatsu Miniature Spectrometer (Hamamatsu, Hertfordshire, UK), with a wavelength range and spectral resolution of 340–850 nm and 15 nm, respectively, with beam steering provided by New Scale Technologies DK-M3-RS-U-2M-20-L, (New York, NY, USA), Rotary Mirrors (Figure 2). These components are commercially available and easily programmed to suit the proposed application. Ray trace modelling was performed to estimate the theoretical instantaneous field of view (IFOV) of the single pixel upon the target. This was calculated to be approximately 2.50 mm × 4.90 mm for a 95% energy enclosure, and it includes the diffraction limit of the spectrometer. This also accounts for aberrations resulting from the use of a low-cost single lens. The maximum angle of the mirror system is ±20° providing a total field of view (TFOV) of 36.4 cm for an image; however, the ability of the optics to form a focused image limits the TFOV below this maximum value. No additional coupling optics were used before the spectrometer.

A bi-directional raster scanning pattern is implemented to capture each pixel in a scene, with beam steering provided by the rotary mirror system. The mirror system comprises a short mirror of dimensions 3 mm × 5 mm × 0.4 mm, and a long mirror of dimensions 11 mm × 5 mm × 0.4 mm. Image resolution is user defined and, therefore, configurable to the requirements of the proposed application. Exposure time per pixel can also be configured by the operator to best fit the illumination of the target. Whilst increases to the exposure time per pixel and the resolution of a scene will help to provide clearer imagery, there remains an inevitable trade-off with the overall scan time required.

The data collected are then loaded into a specifically designed LabVIEW programme which converts the collected one-dimensional data array into the data cube format. Furthermore, the imager is controlled with LabVIEW software where operator input through a computer terminal can determine acquisition parameters, such as the required dimensions of the scene and the exposure time required per pixel. As the scene is captured, the spectral response can be visualised in real time, highlighting the responses of individual pixels. After scene capture is complete, the operator can use the data cube to scroll through the data captured at different wavelengths. The spectral response from specific pixels can also be visualised in a separate graph to allow for more detailed analysis. To remove the spectral influence of illumination and sensor sources, the following analysis was implemented:

(1) $$Ratio=\frac{S_{target}-S_{dark}}{S_{white}-S_{dark}}=\frac{R_{target}}{R_{white}}\approx R_{target},$$

where *S* represents the measured signal and *R* represents the reflectance spectrum. The subscripts target, dark, and white represent the imaged object, dark offsets, and white reference, respectively. Note, to provide a value for *R_white_*, a reference “orb” was three-dimensionally printed and coated with Edmund Optics white reflectance coating (Stock #83–890). This coating was applied using an airbrush to ensure uniform coverage across the object and is specified to provide >97% reflectivity from 350–850 nm. The “orb”, a ca. 60-mm-diameter sphere, was used in place of a target object to obtain the white reflectance value. The digital equivalent of a resistor–capacitor (RC) filter was then applied to the spectral data within LabVIEW as a smoothing factor to minimise the influence of excess noise. This correction was applied to all datasets discussed in this article with displayed data showing scaled reflectance as calculated in Equation (1). The imager is capable of capturing a variety of scenes, making it practical for a versatile range of applications, and it allows for real-time monitoring and decision-making, which is of significant benefit to a variety of measurement and monitoring applications.

**Figure 3:**
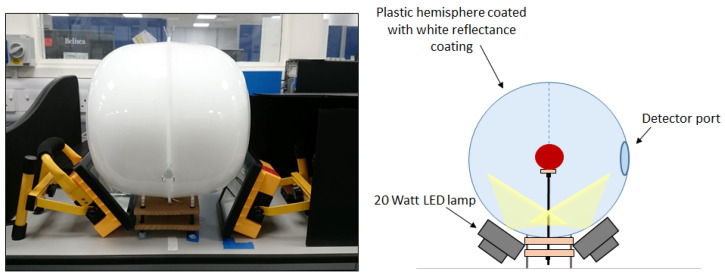
Low-cost integrating sphere set-up for image capture. Schematic not to scale.

**Figure 4:**
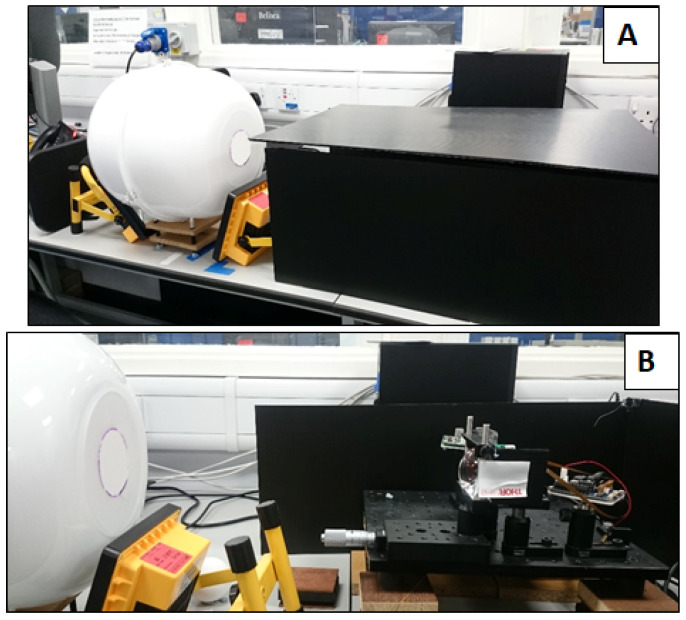
The current set-up of the hyperspectral imager: (**A**) displays the true set-up during image capture, with the hyperspectral imager covered by a dark box, while (**B**) displays the alignment between the sphere and the hyperspectral imager with the dark box removed.

Directional scatter across a target object can often result in the manifestation of bright spots and shadowing across an acquired image. To prevent this, during our measurements, the object to be imaged was placed within a low-cost integrating sphere (Figure 3). This sphere comprises two plastic hemispheres, ca. 30 cm in diameter, coated with the same Edmund Optics white reflectance coating as applied to the “orb”. A detector port is present for the hyperspectral imager, and two lighting ports allow for object illumination provided by two 20-W light-emitting diode (LED) lamps angled to prevent the direct illumination of the sensor. The inclusion of the integrating sphere provides uniform illumination across the object surface, minimising the shadow and directional scattering resulting from a three-dimensional surface. The object is diffusely illuminated by the sphere, which ensures that any variations observed are a result of variations within the object and not resultant from favourable lighting e.g., bright spots and/or shadows. The hyperspectral imager is covered during image acquisition to prevent the interference of ambient stray light during image capture. In its current format (Figure 4), the low-cost hyperspectral imager is suitable for laboratory-based, bench-top image acquisition. It is capable of a variety of image capture applications, making it a valuable laboratory measurement tool.

To assess the optical power reflected from the surface of the target objects and, ultimately, the power represented by each pixel of the image, a radiometric measurement of the power reflected from the surface of the white reference “orb” was measured. This was measured using the silicon photodiode-based radiometer as described in Zhu et al., [11]. For this measurement, the RG850 long-pass filter was removed to allow light corresponding to the wavelength range of the imager to be measured. It was replaced in the sight path with a narrow bandpass filter from Thorlabs (Stock #FB550–10, Ely, UK), centred on 550 nm with a full width half maximum of 10 nm. The photocurrent was measured with the radiometer sighted upon the orb with and without the narrow bandpass filter in place. From these data and the spectral properties of the radiometer and filter, the reflected optical power collected by the radiometer was calculated to be 13.8 nW. Given that the FOV of the radiometer represented a circular area of approximately 14 mm in diameter upon the target, and assuming diffuse reflections, the reflected power per unit area was calculated to be approximately 89.6 μW/m^2^. Therefore, given that each pixel of the hyperspectral imager represents an area of 12.25 × 10^−6^ m^2^, the total power collected per pixel of the imager is approximately 1.10 nW for the white reference “orb”. This calibration enables samples from different sources to be accurately compared, significantly increasing the imager’s usability. Furthermore, as these measurements are traceable to the watt, it provides a degree of precision that would not otherwise be available. Traceability was by means of a certified, calibrated radiation thermometer (AMETEK Land Cyclops C100, Land Instruments, Dronfield, UK) that was used in tandem with an approximate blackbody furnace (AMETEK Land R1500P Land Instruments, Dronfield, UK) to calibrate the aforementioned radiometer that, in turn, was used to calibrate our hyperspectral imaging system. The origin of the traceability was the United Kingdom (UK)’s National Measurement Institute (NPL, Teddington, UK).

## 3. Results and Discussion

### 3.1. Environmental Applications

In order to demonstrate our imager’s capabilities, we tested it within a variety of laboratory-based applications. The applications discussed below were selected as they represent areas of research where hyperspectral datasets were shown to be beneficial; however, the current literature suggests that they are lacking in low-cost, accessible hyperspectral imagers at this time. By demonstrating our imager’s capabilities in these domains, we aim to introduce robust low-cost hyperspectral systems for ongoing development in these settings.

#### 3.1.1. Fruit Quality Identification

Hyperspectral imaging was shown to be an effective, non-destructive means of quality assessment for a variety of food categories [12,13,14,15], providing an accurate early detection method for product deterioration [16], which may not be so easily recognised with traditional analysis methods [17,18,19]. Whilst qualitative assessments in this field are typically completed using features present within the near-infrared portion of the electromagnetic spectrum [17,20,21], research showed that absorption features present within the visible spectrum can also be used as a low-cost alternative means of quality control [17,22,23,24]. Similar experiments were completed by, for example Hossain et al. [22] and Das et al. [25] to test a variety of smartphone spectrometers [26]; however, to the best of our knowledge, this represents the first test of a low-cost hyperspectral imager in this capacity.

**Figure 5:**
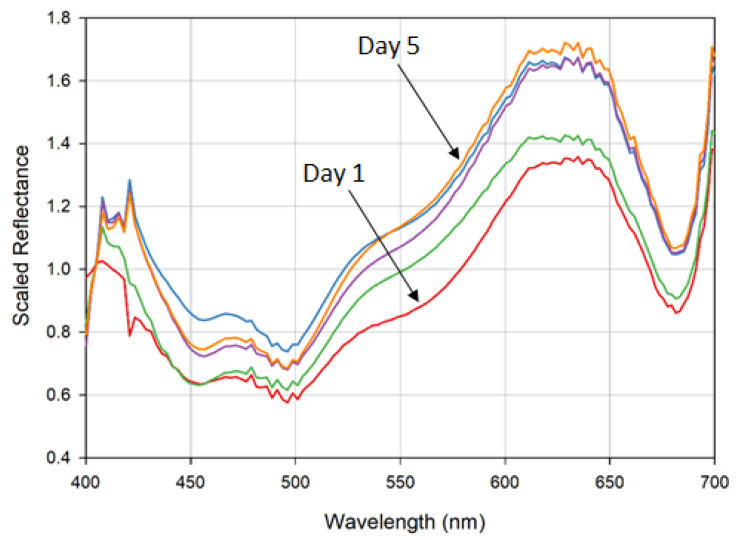
Spectral reflectance of a healthy apple measured over a five-day period, highlighting the changes in pigments that occur during the ripening process. Note the absorption features present at ca. 550 nm, ca. 650 nm, and ca. 675 nm. Scaled reflectance is representative of *R_Target_* in Equation (1).

We focused on two key areas within this field: changes in spectral reflectance as the fruit ages, and the development and identification of fruit bruising. The fruits were imaged over the course of five days in order to detect any pigment variations or bruise development in the affected fruits. During image acquisition, each fruit was placed within the integrating sphere and illuminated as described in Section 2. A 128 × 128 pixel scan with an exposure time of 15 ms per pixel was then acquired. These parameters were selected as they represented a suitable balance between image quality and acquisition speed, allowing for the collection of images with appropriate spatial resolution to define target object features with minimal time restraints. When image capture was not taking place, the fruits were stored under ambient light within the laboratory. From the observed data, the pixels that make up the target object were then averaged to produce one comparable response from each fruit measured, allowing for comparisons to be drawn between different fruits and/or different days over the measurement period. Figure 5 displays the reflectance spectrum of a healthy apple over the course of the five-day measurement period.

As the fruit ages, an increase in reflectance is expected to occur as a result of the breakdown and transformation of fruit pigments during the ripening process [27,28]. This gradual increase in reflectance over time is clearly displayed in the spectra collected using the low-cost hyperspectral imager, correlating well with the results of previous research. Furthermore, the absorption features of the fruit pigments are also clearly visible within this data, notably, the stagnation at ca. 550 nm which can be attributed to anthocyanin absorption, a shoulder at ca. 650 nm related to chlorophyll b absorption, and the distinct loss in reflectance at ca. 675 nm highlighting the presence of chlorophyll a [22,24]. Whilst the peaks and troughs present within the blue spectrum in this figure may be associated with variations in carotenoids within the fruit [24], the features are not distinct enough in this dataset to pinpoint. It is, therefore, believed that these fluctuations are anomalies resulting from noise located in this section of the dataset.

**Figure 6:**
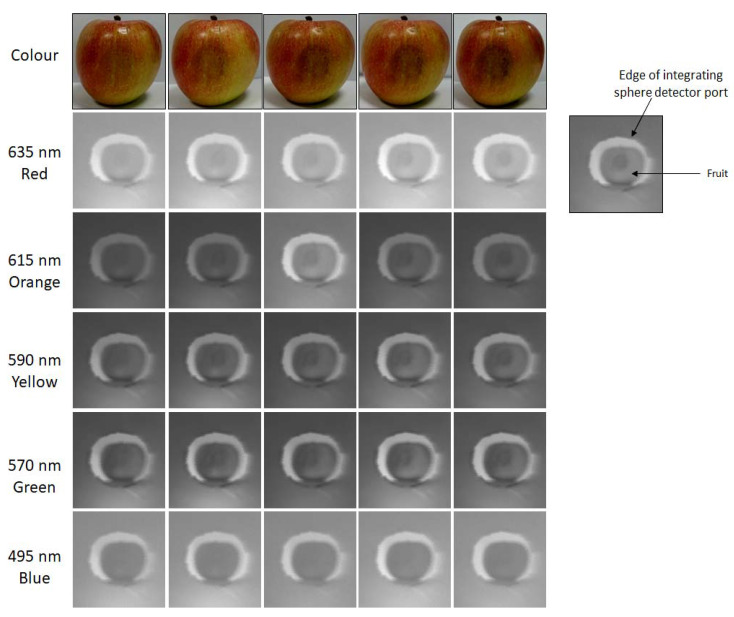
Bruise development over the measurement period: comparison between colour and hyperspectral datasets captured with a 128 × 128 pixel scene at 15 ms exposure per pixel. Note the varying levels of detection at different wavelengths.

The incorporation of hyperspectral analysis to fruit quality control was shown to provide significant improvements in the accurate identification of poor-quality, damaged produce [15,29,30]. These improvements are further illustrated in the data collected by this study. It was established that, particularly during the early stages, bruise identification can be extremely difficult due to the near invisibility of initial symptoms [19,31]. Figure 6 highlights the varying degrees of bruise detection across the different wavelengths. Bruising is much more obvious at wavelengths across the red–green spectrum due to the increased reflectivity of the healthy tissues at these wavelengths, which emphasises the decreased reflectance of the damaged tissue. Conversely, bruising remains almost invisible in the blue portion of the spectrum due to the generally lower reflectance of fruit tissues at these wavelengths, making the apple appear more homogeneous. Furthermore, the detection of bruising on fruits with darker pigmentations can remain unobvious for extended time periods using standard colour image techniques, increasing the likelihood of deterioration of additional produce within a batch. However, with the use of hyperspectral datasets, which can pinpoint individual wavelength responses, these bruises can be identified much more efficiently [15,32]. With the introduction of low-cost hyperspectral measurement methods, such as the imager discussed in this article, this level of high-quality produce analysis can become more readily available, providing a substantial advantage to the industry through the introduction of affordable hyperspectral sensors.

#### 3.1.2. Volcanic Rock Mineralogy

The characterisation of the surface spectral reflectance of volcanic rocks is an important area of research that is seeing a recent increase in popularity [33]. Traditionally, characterisation of these materials is completed through satellite-based remote sensing; however, this is limited by the comparatively low spatial resolution of hyperspectral satellite data, which results in significant spectral mixing [3,33]. Laboratory-based measurements, therefore, represent a suitable alternative. Previous studies, e.g., Abrams et al. [34], Li et al. [35], Aufaristama et al. [36], and Amici et al. [37], completed spectral analyses on a variety of volcanic rocks providing substantial information about their variable spectral responses. We, therefore, tested our low-cost hyperspectral imager’s abilities in this discipline with an outlook to producing a version that can be used in a field setting in future.

**Figure 7:**
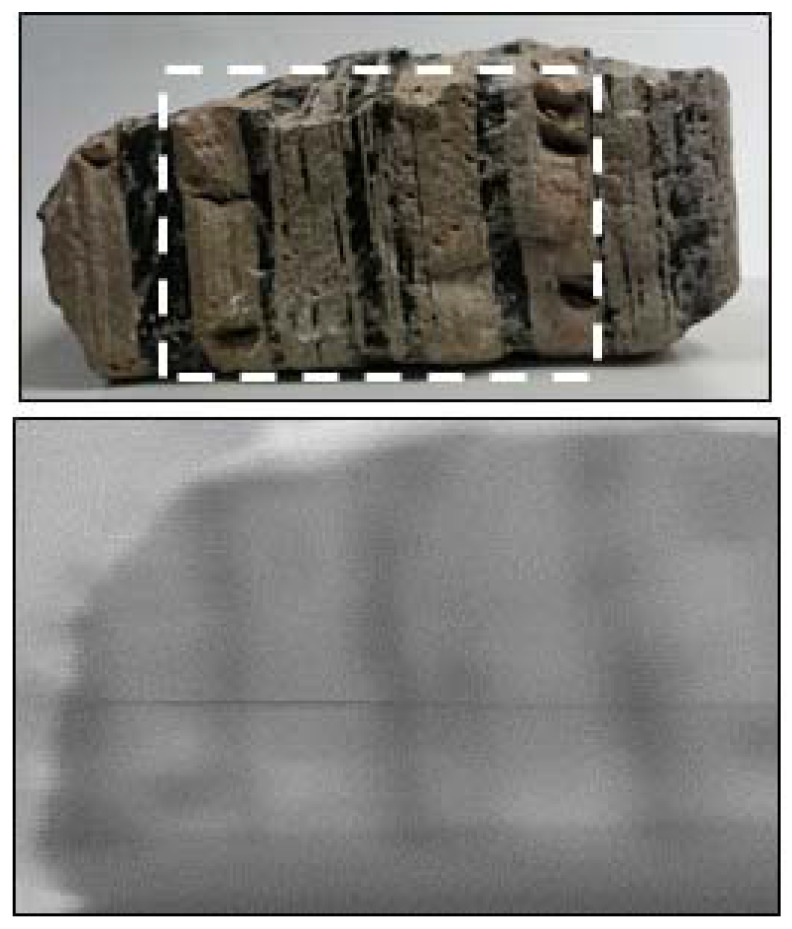
Example image captured using the low-cost hyperspectral imager displaying the presence of flow banding. Hyperspectral image taken from 613 nm of a 256 × 256 pixel scan.

A variety of volcanic rocks of differing crystal size were imaged using the hyperspectral imager set-up. A 128 × 128 pixel scan was taken of each rock with an additional 256 × 256 pixel scan which provided a more detailed view of different rock features. These images were acquired with an exposure time of 25 ms. This longer exposure was required due to the more limited reflectance of these objects. Within the rocks sampled, several contained varying crystals, such as flow banding, to determine whether the low-cost imager would be capable of differentiating these variable features. Figure 7 displays an example image recorded of an ash tuff with obsidian flow banding. The hyperspectral data demonstrate that this imager is capable of distinguishing between the different rock features present within the sample. Image clarity could be improved with the upgrade of the current lens system as the currently installed lens represents a basic set-up. An improved optical system, such as a custom design featuring multiple lenses, would be capable of mitigating aberrations, achieving greater image clarity. However, the inclusion of such a system would result in a significant increase in incurred costs and, therefore, does not fit with the aims of this low-cost design. In its current format, it is clear that the imager is capable of identifying different rock features within a sample, highlighting its potential for growth and development in this field.

**Figure 8:**
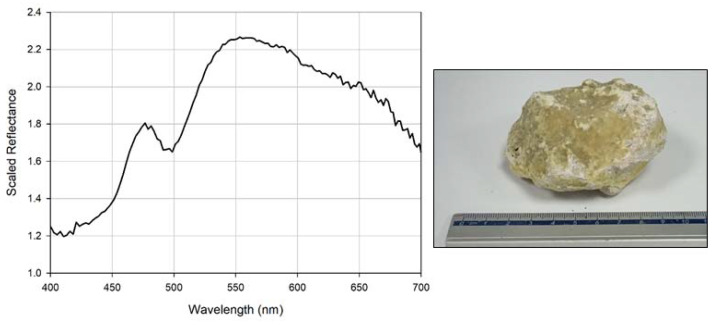
Observed spectral reflectance for the sulphur sample (**right**); note the significant increase in reflectance observed from ca. 500 nm in the spectral data (**left**). Scaled reflectance is representative of *R_Target_* in Equation (1).

**Figure 9:**
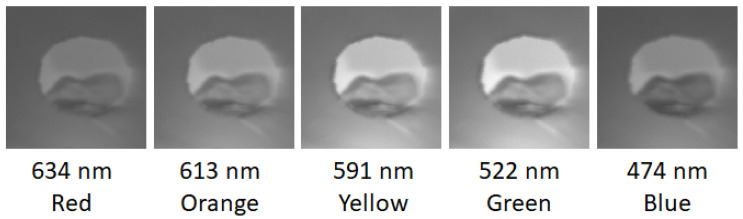
Variations in reflectance across the hyperspectral data for the sulphur target. Images taken from a 128 × 128 pixel scan.

Whilst volcanic rocks, such as basalt, obsidian, and andesite, typically display low reflectance values within the visible spectrum, with minor variations resulting from oxidization and/or vegetation growth [34,35,37], minerals, such as sulphur display much more distinctive reflectance curves [38,39]. Figure 8 shows the reflectance curve obtained from a sulphur rock imaged using the low-cost hyperspectral imager. This figure clearly displays the expected increase in reflectance from ca. 500 nm that is observed with the sulphur mineral, where variations in this curve are believed to relate to the obvious variations across the surface of the rock sample used. Furthermore, Figure 9 displays some of the hyperspectral data collected during image acquisition of the sulphur rock. This figure demonstrates the brightest responses present in the yellow and green regions of the spectrum, correlating well with the observed spectral reflectance curve.

The ability to differentiate between the spectral reflectance and identify crystal variations in volcanic rocks is of significant benefit to the research community, helping to improve our knowledge of these changeable environments, as well as providing planetary analogues for ongoing solar system exploration missions [37,40,41,42]. The results derived from this imager highlight its proficiency within this field. Future work will look to better develop the set-up in order to allow it to be accurately implemented in a variety of field environments as a low-cost portable imager.

#### 3.1.3. Tooth Shade Determination

**Figure 10:**
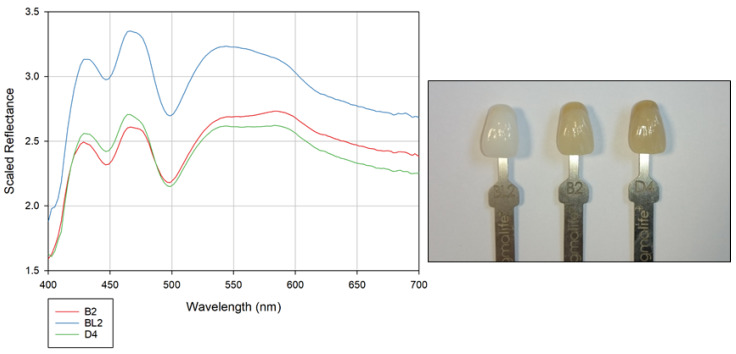
Spectral response across the visible spectrum for three dental shade tabs of varying shades. Scaled reflectance is representative of *R_Target_* in Equation (1).

Tooth aesthetics plays an important role in the appearance of the mouth, and factors such as tooth form, shape, and colour, together with the shape of the dental arches, contribute to this [43]. Accurate tooth colour matching is, therefore, an important consideration when achieving good-quality dentistry. Matching the colour of synthetic tooth replacements to existing teeth can be a challenge, especially for single-unit replacements. Belser et al. [44], proposed a White Aesthetic Score index (WES) to address this issue in single-tooth implants. This index also takes into account other important factors, such as tooth form, volume, outline, surface texture, and translucency. The WES index, whilst originally devised for use in implantology, lends itself nicely to all aesthetically driven restorations, and it encapsulates well the different factors that need to be considered regarding aesthetic dental restorations. There are different commercially available systems to help the dentist with regard to colour matching; these include visual shade guide systems such as the samples used below (Figure 10), comprising multiple handled tabs of differing hue, value, and chroma, as well as automatic shade determination devices such as colourimeters, digital imaging devices, and spectrophotometers.

Visual determination was shown to be very subjective, and is affected by factors such as the quality of background illumination, degree of hydration of the tooth surface, and eye fatigue. [45,46]. Automatic shade determination technology, therefore, advanced considerably in the last 20–30 years and, while not wholly accurate, it was shown to have good reproducibility and reliability [47,48,49]. Spectrophotometry was shown to be the most precise and accurate method of visual determination; whilst inconsistent, it is not necessarily less precise than colourimetric methods [48,50]. Hyperspectral imaging has the potential to provide a means of more reliable shade determination. We, therefore, chose to test the low-cost imager’s capabilities in this field.

We imaged three dental tabs of different shades (BL2, B2, and D4) using the hyperspectral imager set-up described above. These images were captured at 256 × 256 pixels due to the small size of the target objects, with an exposure time of 15 ms per pixel. Figure 10 displays the spectral reflectance responses gathered for each shade. In this figure, the varying shades of the tabs are clearly demonstrated; BL2, the tab with the lightest shading, can be seen to produce the highest level of reflectance across all wavelengths measured, with B2 providing a slightly brighter reflectance than D4, as would be expected given the relative shading of these tabs. Furthermore, the spectral response of both B2 and D4 also displays a slight increase in reflectance at ca. 590 nm, whereas BL2 displays a slight drop in reflectance in this region. This more pronounced response from B2 and D4 at this location correlates well with their more yellow colouring compared to BL2. Finally, the spectral responses present in the blue portion of the spectrum could be a result of fluorescing in this region due to its proximity to the ultraviolet spectrum. These results demonstrate the hyperspectral imager’s proficiency in tooth shade determination, as it can capably identify the different shades presented to it. This suggests that the imager has significant ongoing potential in this field of study.

## 4. Conclusions

This article introduced a laboratory-based hyperspectral imager developed from low-cost, commercially available components. We provided an imager calibration enabling the accurate comparison of different samples with traceability to the watt, and we demonstrated the imager’s proficiency within several applications, highlighting it to be a valuable, low-cost laboratory measurement tool capable of both quantitative and qualitative hyperspectral measurements. Its current format allows it to complete bench-top measurements; however, it possesses significant potential for further development as a portable, low-cost hyperspectral imager, with an outlook to incorporation in future field deployments. This imager represents an initial development in accessible hyperspectral technologies, providing a basis for future improvements. The continued development of these low-cost devices is of significant importance to a variety of laboratory- and field-based applications. Their ability to provide accurate hyperspectral measurements at a fraction of the cost of current systems allows for increased opportunities to gain a better understanding of the processes and products that influence a variety of environmental settings.
